# A hybrid qPCR/SNP array approach allows cost efficient assessment of KIR gene copy numbers in large samples

**DOI:** 10.1186/1471-2164-15-274

**Published:** 2014-04-11

**Authors:** Nikolas Pontikos, Deborah J Smyth, Helen Schuilenburg, Joanna MM Howson, Neil M Walker, Oliver S Burren, Hui Guo, Suna Onengut-Gumuscu, Wei-Min Chen, Patrick Concannon, Stephen S Rich, Jyothi Jayaraman, Wei Jiang, James A Traherne, John Trowsdale, John A Todd, Chris Wallace

**Affiliations:** 1 JDRF/Wellcome Trust Diabetes and Inflammation Laboratory, Cambridge Institute for Medical Research, University of Cambridge, Wellcome Trust/MRC Building, CB2 0XY, Cambridge, UK; 2 Cardiovascular Epidemiology Unit, Department of Public Health and Primary Care, University of Cambridge, Strangeways Research Laboratory, CB1 8RN, Cambridge, UK; 3 Center for Public Health Genomics, University of Virginia, 22908-0717, Charlottesville, Virginia, USA; 4 University of Florida Genetics Institute, 32610-3610, Gainesville, Florida, USA; 5 Division of Immunology, Department of Pathology, University of Cambridge, Tennis Court Road, CB2 1QP, Cambridge, UK; 6 Cambridge Institute for Medical Research, University of Cambridge, Wellcome Trust/MRC Building, CB2 0XY, Cambridge, UK; 7 MRC Biostatistics Unit, Institute of Public Health, University Forvie Site, Robinson Way, CB2 0SR, Cambridge, UK

**Keywords:** KIR3DL1, KIR3DS1, KIR2DS4, KIR2DL3, KIR2DL5, KIR2DS5, KIR2DS1, HLA-Bw4, CNV, qPCR, ImmunoChip, KIR, Imputation, T1D

## Abstract

**Background:**

Killer Immunoglobulin-like Receptors (KIRs) are surface receptors of natural killer cells that bind to their corresponding Human Leukocyte Antigen (HLA) class I ligands, making them interesting candidate genes for HLA-associated autoimmune diseases, including type 1 diabetes (T1D). However, allelic and copy number variation in the KIR region effectively mask it from standard genome-wide association studies: single nucleotide polymorphism (SNP) probes targeting the region are often discarded by standard genotype callers since they exhibit variable cluster numbers. Quantitative Polymerase Chain Reaction (qPCR) assays address this issue. However, their cost is prohibitive at the sample sizes required for detecting effects typically observed in complex genetic diseases.

**Results:**

We propose a more powerful and cost-effective alternative, which combines signals from SNPs with more than three clusters found in existing datasets, with qPCR on a subset of samples. First, we showed that noise and batch effects in multiplexed qPCR assays are addressed through normalisation and simultaneous copy number calling of multiple genes. Then, we used supervised classification to impute copy numbers of specific KIR genes from SNP signals. We applied this method to assess copy number variation in two KIR genes, *KIR3DL1* and *KIR3DS1*, which are suitable candidates for T1D susceptibility since they encode the only KIR molecules known to bind with HLA-Bw4 epitopes. We find no association between *KIR3DL1/3DS1* copy number and T1D in 6744 cases and 5362 controls; a sample size twenty-fold larger than in any previous KIR association study. Due to our sample size, we can exclude odds ratios larger than 1.1 for the common *KIR3DL1/3DS1* copy number groups at the 5% significance level.

**Conclusion:**

We found no evidence of association of *KIR3DL1/3DS1* copy number with T1D, either overall or dependent on HLA-Bw4 epitope. Five other KIR genes, *KIR2DS4*, *KIR2DL3*, *KIR2DL5*, *KIR2DS5* and *KIR2DS1*, in high linkage disequilibrium with *KIR3DL1* and *KIR3DS1*, are also unlikely to be significantly associated. Our approach could potentially be applied to other KIR genes to allow cost effective assaying of gene copy number in large samples.

## Background

Killer Immunoglobulin-like Receptors (KIRs) are transmembrane glycoproteins expressed by natural killer cells and subsets of T cells. The KIR region lies in a 150 kb gene cluster located within the 1 Mb Leukocyte Receptor Complex on chr19q13.4. The region exhibits great haplotype and copy number diversity, which has prevented complete assessment of the KIR genes in standard genome-wide association studies (GWAS), despite their strong candidacy for immune-related traits. Targeted quantitative Polymerase Chain Reaction (qPCR) assays have been used to detect presence or absence of individual KIR genes and more recently, determine copy numbers [1]. Nevertheless these remain expensive and labour intensive compared to SNP arrays.

We show that SNPs often discarded in GWAS, because they exhibit non-typical number of genotype clusters, can be informative of KIR gene copy numbers. By applying supervised classification, we are able to use qPCR results in a modest number of samples to impute copy numbers into a larger sample for which SNP array signals are available. We illustrate this method by applying it to two genes in the KIR complex, *KIR3DL1* and *KIR3DS1*, which are suitable candidates for T1D association due to their interaction with HLA class I molecules. Specifically, the KIR3DL1 protein is known to interact with the HLA class I allotypes that contain the HLA-Bw4 serological epitope [2,3], whereas the protein encoded by *KIR3DS1*, which shares 97% sequence similarity to *KIR3DL1*, is thought to bind the more restrictive HLA-Bw4-80I epitope subset [4]. The grouping of HLA-A and HLA-B alleles according to HLA-Bw4 serological epitope [5] is given in Additional file 1: Table S1 and includes several HLA class I alleles which are associated with T1D risk after conditioning on the major HLA class II effects [6,7]. To date, *KIR3DL1/3DS1* association with T1D has only been studied using qPCR assays in limited sample sizes, which assess presence or absence of each KIR gene [8].

We used qPCR copy number calls in 1474 samples as a training set, and imputed copy number in a further 12106 samples from raw genotyping signals in SNP array probes targeting the KIR region. We thus tested association of *KIR3DL1/3DS1* copy number with T1D, either directly, or through interaction with HLA-Bw4. To the best of our knowledge, the sample size of our study is twenty-fold larger than any previous study of *KIR3DL1/3DS1* in T1D, and the first to test copy number variation rather than simply presence or absence [8].

The hybrid method we advocate, leverages the information available from targeted qPCR assays in modest samples to the level of sample coverage required for modern, well-powered genetic studies. It has the potential to be applied to other genes in the KIR region or, indeed, to other chromosome regions that exhibit similar copy number variation and sequence complexity.

## Methods

### Subjects

DNA was available from 12106 individuals: 6744 cases (age of diagnosis less than 17) from the Genetic Resource Investigating Diabetes (GRID) cohort, and 5362 controls from the British 1958 Birth Cohort (1958BC). All subjects were of white European ancestry with written informed consent and Ethics Committee/Institutional Review Board approval. The GRID cohort was approved by the Cambridgeshire 4 Research Ethics Committee, study title “Developing targets for Diabetes prevention by the study of the genetics of Type 1 diabetes” (ref 00/5/044). The 1958BC cohort was approved by the North West Ethics Committee, study title “1958 Birth Cohort Tissue Bank” (ref 09/H1010/12). The use of these samples was approved by the Cambridgeshire 2 Research Ethics Committee, study title “Investigating Genes and Phenotypes associated with Type 1 Diabetes” (ref 08/H0308/153).

Ancestry was confirmed by PCA analysis of earlier GWAS data in these samples [9]. The DNA for the cases and controls was prepared using the same protocols in Cambridge and in Bristol respectively. All samples were cell-line derived.

HLA genotypes were available on a subset of 5603 individuals, 2922 cases and 2681 controls. HLA-A and HLA-B genes were typed at four-digit allele resolution using Dynal RELI SSO assays (Invitrogen, Paisley, U.K.) (Additional file 1: Table S3). The epitope classification of HLA-A and HLA-B alleles is given in Additional file 1: Table S1. All 12106 samples have been genotyped using ImmunoChip, a custom Illumina 200K Infinium high-density SNP array [10], according to the manufacturer’s protocol, processed at the University of Virginia in Charlottesville, USA. A random subset of 1629 samples, 816 cases and 813 controls, for which HLA genotype was available, were selected for qPCR. These samples were arrayed on bespoke 96-well plates, randomised half cases, half controls. The source plates were chosen as those containing samples with the most HLA typing available at the time (2009).

### Design of multiplexed qPCR *KIR3DL1/3DS1* copy number assay

The qPCR platform used was the LightCycler 480 Real-Time PCR Instrument on which we ran eighteen 384-well plates and four repeated plates. The 1629 samples, 816 cases and 813 controls, selected for qPCR were arrayed evenly, half-cases, half-controls, across the plates. On the four repeated plates, 310 samples were arrayed (Additional file 1: Figure S3). On each plate, every sample was replicated across four neighbouring wells, resulting in a maximum of 96 samples per plate. All plates, except for one, contained four repeated calibrator samples of known *KIR3DL1/3DS1* copy number that included, two samples with *KIR3DS1*-*KIR3DL1* copy number 1-2 and two samples with *KIR3DS1*-*KIR3DL1* copy number 2-1. These are represented in Additional file 1: Figure S2 as black points. To detect contamination, each plate also included one water well. Four plates were analysed in duplicate in order to assess reproducibility (Additional file 1: Figure S3).

The qPCR probes and forward/reverse primers were carefully designed, in collaboration with Jiang et al. [1], to target and amplify most known *KIR3DL1* and *KIR3DS1* alleles, as well as the reference gene *STAT6*, known to always be present in two copies. The probe and primer sequences are summarised in Additional file 1: Table S2.

Each qPCR well was multiplexed, so that the copy numbers of *KIR3DL1*, *KIR3DS1* and *STAT6* were simultaneously assayed as part of the same qPCR reaction. To allow for this, the probes were conjugated with three distinct dyes: Fam for *KIR3DS1*, Cy5 for *KIR3DL1* and DFO for *STAT6*.

Each qPCR well reaction was prepared with 2 *μ*l of DNA at 5 ng *μ*l^-1^ and 5 *μ*l of Quantifast Multiplex PCR mastermix (0.25 *μ*l primer mix, 0.045 *μ*l probe mix and 4.705 *μ*l of water). The qPCR conditions were 95°C for 5 min, followed by 40 cycles at 95°C for 15 s and 66°C for 50 s. Data was collected at 66°C.

### Quality control and normalisation of the qPCR data

The experiment files exported from the LightCycler gave us three crossingpoint (Ct) values per well, one for each of the dye-DNA conjugates. The Ct value is representative of the number of qPCR cycles required for the dye-DNA conjugate to be sufficiently amplified for the fluorescence to cross the detection threshold. Hence a larger Ct value usually implies a smaller underlying copy number. For each well, by subtracting the Ct of Fam-*KIR3DL1* and Cy5-*KIR3DS1* from the Ct value of the DFO-*STAT6*, the reference dye-DNA conjugate, we obtained the baseline relative *Δ*Ct value for *KIR3DS1* and *KIR3DL1*. Then for each sample, we took the median over the four replicate wells to obtain per sample, *KIR3DS1* and *KIR3DL1Δ*Ct values (Additional file 1: Figure S1a.b). However, certain wells did not yield a *STAT6* Ct value since the detection threshold was not crossed within the 40 PCR cycles. We found 64 samples that did not yield a DFO-*STAT6* Ct reading in all four well replicates and these were excluded in the first step of our quality control (QC). Visual inspection of the *KIR3DL1* and *KIR3DS1Δ*Ct distributions by plate led us to drop plate 22 (highlighted in Additional file 1: Figure S1a.b) as it appeared to contain numerous outliers. This resulted in a further 91 samples being dropped as part of our QC. Following QC, we were left with 1474 unique samples, 747 cases and 727 controls, over 17 plates.

Individual distributions of *KIR3DS1* and *KIR3DL1Δ*Ct were not aligned between plates (Additional file 1: Figure S1.c.d), this prevented pooling of all plates for copy number calling. To align the *Δ*Ct distributions across the 17 plates, we first applied the k-medoids algorithm within each plate to the *Δ*Ct *KIR3DS1* and *KIR3DL1* separately to identify the location of the most distinguishable copy number groups, one and two copies. We then normalised across plates by a linear transformation so that the median *Δ*Ct of the two groups mapped to 1 and 2 across all 17 plates (Additional file 1: Figure S1.e.f). After normalisation, negative *Δ*Ct values were assigned to zero to reflect their expected copy number state.

Following QC and normalisation, samples which were repeated across different plates showed good reproducibility (Additional file 1: Figure S3). These were summarised by the median of their repeated value.

### Copy number calling and multiple imputation in the subset of samples with qPCR data

Samples with three or more missing Fam-*KIR3DL1* or Cy5-*KIR3DS1* Ct values out of the four well replicates, were assigned to zero copies of *KIR3DL1* or *KIR3DS1* respectively.

**Figure 1:**
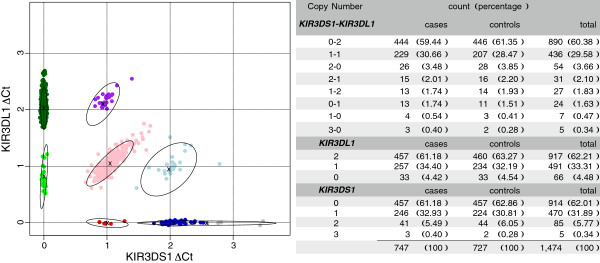
**Bivariate copy number calling of*KIR3DL1/3DS1* from qPCR*Δ*Ct.** On the left, the median normalised *Δ*Ct values for *KIR3DS1* and *KIR3DL1* are shown with the results of clustering into the eight copy number groups coloured according to the group with the highest posterior probability. The three most common *KIR3DS1-KIR3DL1* copy number groups are the ones with a total copy number of two: 0-2 (dark green), 1-1 (pink) and 2-0 (dark blue). The ellipses delimit the 95^*th*^ percentile. On the right, the counts of the most probable copy number groups are shown for cases and controls.

For the remainder of the samples, copy number calling was done jointly on *KIR3DL1* and *KIR3DS1* using unsupervised clustering with a finite mixture model. We called copy number groups by fitting a mixture of eight bivariate Gaussian distributions to exploit the notable correlation between the normalised *KIR3DS1* and *KIR3DL1Δ*Ct values (Additional file 1: Figure S2). We allowed for eight *KIR3DS1*-*KIR3DL1* copy number groups: three common groups of two copy numbers (0-2, 1-1, 2-0) and five rarer groups of lower or higher copy numbers (2-1, 1-2, 0-1, 1-0, 3-0) (Figure 1). The mixture was fitted using an EM algorithm [11] with initial parameters calculated from the clusters returned by k-means with centers set to the eight expected locations of the copy number groups. After fitting the mixture model, each sample was assigned eight posterior probabilities of belonging to each of the eight copy number groups, allowing for uncertainty in copy number calling. These posterior probabilities were used to simulate ten plausible *KIR3DS1*-*KIR3DL1* copy number datasets. These ten multiply imputed datasets allowed for statistical analysis to be conducted in parallel and inference to be combined across datasets using the methods described by Little and Rubin (1987) [12] and implemented in the mitools and mice R packages [13,14].

### Copy number imputation into the extended samples

We extended our sample size by using the subset of samples common between the qPCR and SNP datasets, 747 cases and 727 controls, to train a k-nearest neighbour (knn) classifier to predict *KIR3DL1/3DS1* copy number using the *R* and *θ* signals from ImmunoChip SNPs.

Illumina arrays, such as the ImmunoChip, have two fluorescent probes which differ on one base and allow discrimination of biallelic SNPs. The fluorescent intensities are *X* and *Y*, from which are derived the sum, *R* = *X* + *Y*, and the ratio, $\tan(\theta )=\frac{X}{Y}$.

**Figure 2:**
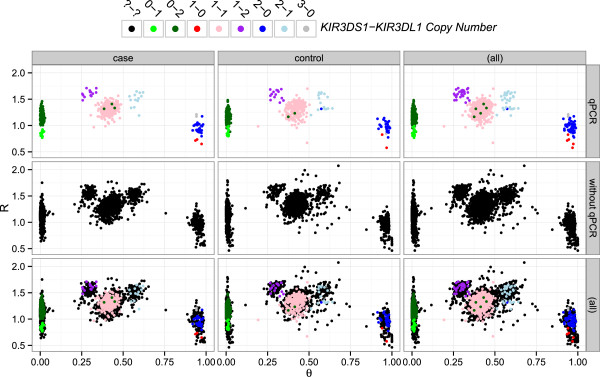
**Overlay of ImmunoChip and qPCR samples for*R* and*θ* at SNP rs592645.** Samples are coloured by the most likely *KIR3DS1-KIR3DL1* copy number group according to the qPCR analysis (see Figure 1). It should be apparent that *R* is representative of the total copy number whereas *θ* relates to the ratio of copies of *KIR3DL1* to *KIR3DS1*. The first and second row split the samples on the availability of qPCR data, and the third row is the overlay of the samples from the first and second row. The first and second column split the samples by case-control status and the third column is the overlay of the samples from the first and second column.

**Figure 3:**
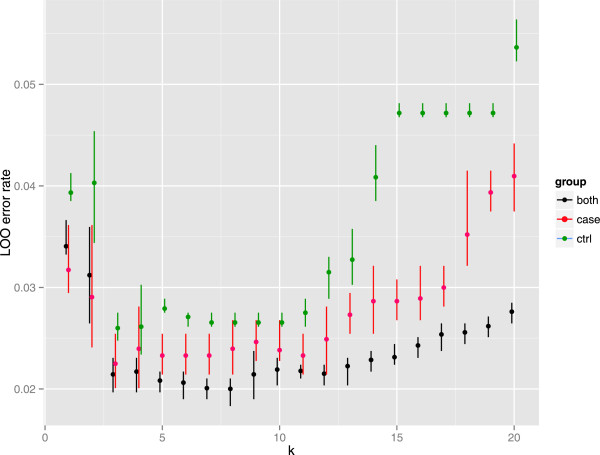
**Leave-one-out crossvalidation error rate for k-nearest neighbour prediction.** Leave-one-out cross validation error rates obtained from k-nearest neighbours (knn) prediction of *KIR3DL1/3DS1* copy numbers from the *R* and *θ* signals of SNP rs592645. Each point shows the proportion of samples for which the knn predicted copy number did not match the qPCR call, averaged over ten multiply imputed qPCR call datasets (using the posterior probabilities from Figure 1). Error bars show the minimum and maximum error rates over the ten multiply imputed datasets. Knn was run in parallel for cases only, controls only and on all samples together. The minimum error rate is achieved for *k*=8 when the prediction uses both cases and controls.

**Figure 4:**
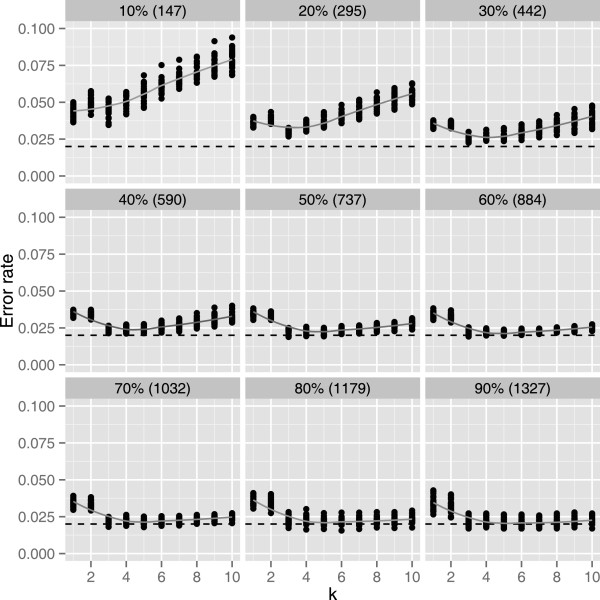
**Error rate of k-nearest neighbour prediction from*R* and*θ* of rs592645 in random subset of samples.** Each panel shows the LOOCV error rates of *KIR3DL1/3DS1* copy number prediction from *R* and *θ* of rs592645 in the remaining unlabeled samples when using a different size subset of the training data. The percentage of the complete training data set and the size of the subset is given in the title of each panel. Each point represents the LOOCV error rate averaged over ten multiply imputed qPCR call datasets (using the posterior probabilities from Figure 1). Smoothing lines show the average over 25 independent random subsets of training data. The black dashed line represent the observed error rate in the complete sample. As the size of the training dataset increases the error rate becomes less sensitive to the choice of the parameter k. Only 295 samples are required to achieve LOOCV error rates <5*%* and 590 for error rates <2.5*%*.

Each of 30 SNPs lying within the *KIR3DL1/3DS1* region, were assessed for association with either *KIR3DL1* or *KIR3DS1* copy number in individual linear regression of copy number against *R* and *θ* (Additional file 1: Table S4). Nineteen SNPs out of 30 were associated (p-value <0.05), nine of which would have failed ImmunoChip QC (Additional file 1: Table S4), with rs592645 the most strongly predictive (Figure 2). We compared running knn with all predictive SNPs or on various subsets, and found rs592645 alone, with *k*=8, minimised the mean leave-one-out cross-validation (LOOCV) error rate over ten multiply imputed qPCR datasets (Figure 3). In each multiply imputed qPCR dataset, all samples were assigned a single imputed copy number group. We also explored the effect of varying the size of the training data set by setting KIR gene copy numbers to missing for a randomly chosen subset of samples and imputing them in the remaining samples (Figure 4).

### Testing for association of *KIR3DL1/3DS1* copy number with T1D

We tested for association of T1D with the predicted copy numbers from the qPCR and SNP datasets using logistic regression. We allowed for uncertainty in the copy number call when estimating individual odds ratios by using the ten multiply imputed datasets generated from the qPCR posterior probabilities [15], and averaging the estimates over those with the R mitools package [13]. We allowed for statistical interaction with HLA-Bw4 by repeating the association test in the subsets of carriers of the target ligand HLA-Bw4 epitopes, HLA-Bw4 for *KIR3DL1* and the putative ligand HLA-Bw4-80I for *KIR3DS1*. We directly tested for interaction with a more powerful case-only *χ*^2^ test [16,17].

## Results and discussion

### Bivariate clustering enables accurate copy number calling in qPCR data

Before normalisation, *Δ*Ct distributions varied across plates preventing simple visual copy number assignment (Additional file 1: Figure S1). After normalisation, samples repeated across different plates showed good reproducibility (Additional file 1: Figure S3). Furthermore, bivariate clustering, on both the *KIR3DS1* and *KIR3DL1Δ*Ct, enabled 1474 samples to be confidently assigned to a single copy number group. Over 99% of all qPCR samples were classified with a posterior probability of copy number group membership greater than 99%. Further, samples of known copy number, the black points in Additional file 1: Figure S2 corresponding to *KIR3DS1*-*KIR3DL1* copy number 2-1 and 1-2, were assigned to the correct copy number group. We allowed for the limited uncertainty in copy number calling, which mostly stems from distinguishing 2-0 from 3-0 (Figure 1), by means of multiple imputation of ten datasets as described in the Methods.

### Imputation into extended samples by integration of SNP and qPCR data

SNP signals, *R* and *θ*, showed strong association with individual copy numbers of *KIR3DL1/3SD1* for 19 of 30 SNPs in the *KIR3DL1* region (Additional file 1: Table S4). The strongest example is shown for SNP rs592645 in Figure 2, in which clusters can be discerned that correspond closely with qPCR derived *KIR3DL1/3SD1* copy numbers.

This figure also illustrates a number of important points regarding using SNP signals for imputation. Firstly, *θ* corresponds to the ratio of copies of *KIR3DL1* to *KIR3DS1*, while *R* corresponds to the total copy number. Secondly, some clusters overlap, particularly along the *R* axis, making them hard to identify without the qPCR data. Consequently, a large proportion of samples were misclassified when we attempted unsupervised clustering using bivariate finite mixture model approaches, first with PlatinumCNV [18], then with our own, mixture of beta-Gaussian distributions, approach. Finally, the clusters are in slightly different positions in cases and controls, reflecting the known sensitivity of genotyping chips to subtle differences in DNA preparation and storage conditions since they were prepared and processed in two different centers [19,20]. Instead, we used the qPCR copy numbers as training data to perform supervised classification with knn on the SNP signals, which does not explicitly rely on the identification of distinct clusters.

We first explored the validity of our imputation approach by means of LOOCV in the samples with qPCR data. We examined using all 19 predictive SNPs, or various subsets, and found optimal knn imputation was achieved with the single most predictive SNP, rs592645 (LOOCV rate = 2.0%). By varying the size of the training data, we suggest that only 295 samples are required to achieve LOOCV error rates <5*%* and 590 for error rates <2.5*%* (Figure 4).

### No evidence of association of *KIR3DL1/3DS1* copy number with T1D

Finally, we tested for association of *KIR3DL1/3DS1* copy number with T1D status. We found no significant evidence of association, in the qPCR data (747 cases and 727 controls), nor in the extended SNP data (6744 cases and 5362 controls), either overall or with any single copy number group (Table 1).

**Table 1:** **Association with T1D tested in the joint copy number group** ***KIR3DS1*-*KIR3DL1* (a), and in the marginal** ***KIR3DL1* (b) and** ***KIR3DS1* (c) copy number groups**

**a)**	**qPCR**					**SNP**				
***KIR3DS1-KIR3DL1***	**Case:control**	**Total**	**OR**	**95% CI**	**p-value**	**Case:control**	**Total**	**OR**	**95% CI**	**p-value**
**0-2**	444:446	890	1.00			4094:3222	7316	1		
**1-1**	229:207	436	1.11	0.88-1.40	0.3673	2050:1628	3678	0.99	0.92-1.07	0.8349
**2-0**	26:28	54	0.92	0.52-1.61	0.7713	229:225	454	0.79	0.65-0.96	0.0193
**2-1**	15:16	31	0.94	0.46-1.93	0.8695	121:101	222	0.92	0.7-1.2	0.5246
**1-2**	13:14	27	0.93	0.43-2.01	0.8587	98:74	172	1.04	0.77-1.42	0.7822
**0-1**	13:11	24	1.19	0.53-2.68	0.6794	116:77	193	1.19	0.89-1.59	0.2535
**1-0**	4:3	7	1.34	0.30-6.02	0.7031	25:21	46	0.94	0.52-1.68	0.8255
**3-0**	3:2	5	1.52	0.27-8.62	0.6369	11:14	25	0.74	0.3-1.82	0.518
**Overall**	747:727	1474			0.9842	6744:5362	12106			0.3552
**b)**	**qPCR**					**SNP**				
***KIR3DL1***	**Case:control**	**Total**	**OR**	**95% CI**	**p-value**	**Case:control**	**Total**	**OR**	**95% CI**	**p-value**
**2**	457:460	917	1.00			4192:3296	7488	1		
**1**	257:234	491	1.11	0.89-1.38	0.3702	2287:1806	4093	0.99	0.92-1.07	0.8883
**0**	33:33	66	1.01	0.61-1.66	0.9795	265:260	525	0.8	0.67-0.96	0.0151
**Overall**	747:727	1474			0.6651	6744:5362	12106			0.0506
**c)**	**qPCR**					**SNP**				
***KIR3DS1***	**Case:control**	**Total**	**OR**	**95% CI**	**p-value**	**Case:control**	**Total**	**OR**	**95% CI**	**p-value**
**0**	457:457	914	1.00			4210:3299	7509	1		
**1**	246:224	470	1.10	0.88-1.37	0.4096	2173:1723	3896	0.99	0.91-1.07	0.7785
**2**	41:44	85	0.94	0.60-1.47	0.7787	350:326	676	0.83	0.71-0.97	0.0212
**3**	3:2	5	1.24	0.21-7.28	0.8084	11:14	25	0.74	0.3-1.82	0.5119
**Overall**	747:727	1474			0.8044	6744:5362	12106			0.1494

No evidence of a significant, joint or marginal, effect was detected in the qPCR dataset, 747 cases and 727 controls, nor in the SNP dataset, 6744 cases and 5362 controls. Case-control counts shown are derived from the most likely copy number assignment across the ten multiply imputed qPCR and SNP datasets. Statistical inference for association is derived from the multiply imputed datasets using the R mitools package [13]. The last row of each table contains the pooled p-value for each association test using the R mice package [14].

By expanding to these large samples, which would be infeasible to genotype directly with qPCR, we are able to exclude odds ratios outside of the range [.92; 1.08] for the common copy number groups with 95% certainty.

We also repeated the association tests in the subset of individuals, carriers of the HLA-Bw4 epitope, and again detected no significant association (Table 2). A disadvantage of subsetting by HLA-Bw4 is that we lose power by greatly reducing the sample size. A more powerful test for interaction between unlinked genes is a case-only test [16]. If there were an interaction between KIR3DL1/3DS1 and HLA-Bw4 then this should be detectable as a difference in *KIR3DL1/3DS1* copy number frequencies across HLA-Bw4 strata in the cases. However, we found no significant evidence for association in either the qPCR or SNP data sets, before or after summarising the KIR copy number by presence/absence to increase power by reducing the degrees of freedom (Table 3).

**Table 2:** **Association with T1D conditional on the presence of the respective HLA-Bw4 epitope, tested in the joint copy number group** ***KIR3DS1*-*KIR3DL1* (a), and in the marginal** ***KIR3DL1* (b) and** ***KIR3DS1* (c) copy number groups**

**a) HLA-Bw4 subset**	**qPCR**					**SNP**				
***KIR3DS1-KIR3DL1***	**Case:control**	**Total**	**OR**	**95% CI**	**p-value**	**Case:control**	**Total**	**OR**	**95% CI**	**p-value**
**0-2**	259:286	545	1.00			1027:1157	2184	1		
**1-1**	123:128	251	1.06	0.79-1.43	0.6976	555:582	1137	1.08	0.93-1.24	0.3119
**2-0**	16:15	31	1.22	0.58-2.57	0.5985	59:88	147	0.76	0.54-1.07	0.1133
**2-1**	7:13	20	0.59	0.23-1.51	0.2754	34:40	74	0.93	0.58-1.48	0.7529
**1-2**	8:8	16	1.10	0.41-2.98	0.8450	24:33	57	0.85	0.5-1.45	0.5502
**0-1**	10:7	17	1.58	0.59-4.20	0.3621	36:24	60	1.69	1-2.85	0.0491
**1-0**	2:1	3	2.21	0.20-24.50	0.5187	7:4	11	1.97	0.58-6.76	0.2793
**3-0**	3:0	3				5:0	5			
**Overall**	428:458	886			0.8978	1747:1928	3675			0.2173
**b) HLA-Bw4 subset**	**qPCR**					**SNP**				
***KIR3DL1***	**Case:control**	**Total**	**OR**	**95% CI**	**p-value**	**Case:control**	**Total**	**OR**	**95% CI**	**p-value**
**2**	267:294	561	1.00			1051:1190	2241	1		
**1**	140:148	288	1.04	0.78-1.38	0.7787	625:646	1271	1.09	0.95-1.26	0.1975
**0**	21:16	37	1.45	0.74-2.83	0.2822	71:92	163	0.88	0.64-1.21	0.4181
**Overall**	428:458	886			0.5563	1747:1928	3675			0.2586
**c) HLA-Bw4-80I subset**	**qPCR**					**SNP**				
***KIR3DS1***	**Case:control**	**Total**	**OR**	**95% CI**	**p-value**	**Case:control**	**Total**	**OR**	**95% CI**	**p-value**
**0**	159:187	346	1.00			649:733	1382	1		
**1**	93:83	176	1.32	0.92-1.90	0.1370	383:366	749	1.18	0.99-1.41	0.0628
**2**	12:14	26	1.01	0.45-2.24	0.9842	61:75	136	0.91	0.64-1.3	0.607
**3**	2:0	2				3:0	3			
**Overall**	266:284	550			0.5209	1096:1174	2270			0.2416

Association is tested in the subset of individuals carriers of an HLA-Bw4 epitope for the joint *KIR3DS1*-*KIR3DL1***(a)** and marginal *KIR3DL1***(b)** copy number groups and, also tested in the subset of individuals carriers of the HLA-Bw4-80I epitope for the marginal *KIR3DS1***(c)** copy number group. Case-control counts shown are derived from the most likely copy number assignment across the ten multiply imputed qPCR and SNP datasets. Statistical inference for association is derived from the multiply imputed datasets using the R mitools package [13]. The last row of each table contains the pooled p-value for each association test using the R mice package [14].

**Table 3:** **Case-only** ***χ***^**2**^** test for interaction between** ***KIR3DS1*-*KIR3DL1* and HLA-Bw4, across the ten multiply imputed qPCR and SNP datasets**

**a)**		**qPCR**		**SNP**	
		**HLA-Bw4**-	**HLA-Bw4**+	**HLA-Bw4**-	**HLA-Bw4**+
**KIR3DS1**-	**KIR3DL1**+	183	269	739	1063
**KIR3DS1**+	**KIR3DL1**-	12	21	40	71
**KIR3DS1**+	**KIR3DL1**+	113	138	396	613
			p-value =0.4094		p-value =0.4235
**b)**		**qPCR**		**SNP**	
		**HLA-Bw4**-	**HLA-Bw4**+	**HLA-Bw4**-	**HLA-Bw4**+
**KIR3DL1**-		12	21	40	71
**KIR3DL1**+		296	407	1135	1676
			p-value =0.5144		p-value =0.3609
**c)**		**qPCR**		**SNP**	
		**HLA-Bw4-80I**-	**HLA-Bw4-80I**+	**HLA-Bw4-80I**-	**HLA-Bw4-80I**+
**KIR3DS1**-		293	159	1153	649
**KIR3DS1**+		159	107	673	447
			p-value =0.4922		p-value =0.0353

Counts in each contingency table are derived from the most likely copy number assignment across the multiply imputed datasets. To reduce the degrees of freedom and improve power, we summarise copy numbers higher or equal to one by presence (+) and zero by absence (-). The pooled p-value of each *χ*^2^ test, across the multiply imputed datasets, is given in the last row of each contingency table. We find no significant association with HLA-Bw4, within cases, in either the joint **(a)** or the marginal **(b)(c)***KIR3DS1*-*KIR3DL1* distributions.

## Conclusion

Regions with great allelic and copy number variation are difficult to properly assess using GWAS. While genome-wide SNP arrays are typically cost effective ways to assay common genetic variation, very polymorphic regions can make the design of SNP probes that bind uniquely to their target region, difficult or impossible. This has resulted in low SNP coverage in the KIR region for the common SNP arrays. The SNPs that do exist on arrays are often discarded during the QC phase of any GWAS because they do not exhibit the expected three clusters. On the other hand, assaying individual genes can prove expensive. For example, the qPCR assays used here to target *KIR3DL1* and *KIR3DS1* cost £12 per sample.

Further, qPCR derived data, despite careful design and multiplexing, remain subject to noise (plate 22 Additional file 1: Figure S1). We ameliorated this through QC and normalisation across plates, and then jointly clustering *KIR3DL1* and *KIR3DS1*, to exploit the correlation between the *Δ*Ct values. The advantage of joint clustering is demonstrated in qPCR plate 10, where noisy cases (Additional file 1: Figure S1.f) are difficult to assign as one or two copies based solely on their *KIR3DL1Δ*Ct, but are much more clearly distinguishable when we also consider their *KIR3DS1Δ*Ct value (Additional file 1: Figure S2).

As receptors for HLA class I molecules, KIR genes are important candidates for T1D and other diseases that associate with HLA variation. However, researchers have been unable to fully assess their candidacy due to lack of coverage in GWAS and the complexity and expense of KIR gene-specific assays. So far, KIR association studies for T1D have involved small samples sizes and have probed for presence/absence of multiple KIR genes whilst ignoring the respective copy numbers of these genes, with mixed results and no consistent pattern of association emerging. However, copy number variation in KIR could be important, as it is a mechanism which gives rise to a large diversity of haplotypes [1]. Our hybrid approach, as outlined in Additional file 1: Figure S4, allowed us to perform the (twenty-fold) largest study of *KIR3DL1/3DS1* copy number in T1D to date, and to test for association in eight of the most frequent copy number groups (Additional file 1: Table S5). In 12106 samples, we found no association of *KIR3DL1/3DS1* copy number with T1D, alone or conditional on presence of the HLA-Bw4 epitope. Our results suggest that, despite the association of certain HLA-A and HLA-B alleles with T1D and the established biological interaction between HLA-Bw4 and *KIR3DL1*, copy number variation in *KIR3DL1/3DS1* is unlikely to have a significant effect on the risk of developing T1D. Other KIR genes that are in high linkage disequilibrium with *KIR3DL1* and *KIR3DS1* are also unlikely to be associated. According to the Allele Frequency Net database [21], these include *KIR2DS4* (97%) and *KIR2DL3* (86%), for *KIR3DL1* and, *KIR2DL5* (81%), *KIR2DS5* (84%) and *KIR2DS1* (92%), for *KIR3DS1* (http://www.allelefrequencies.net/kir6010a.asp). Thus, copy number variation in *KIR3DL1/3DS1* or neighbouring genes is unlikely to be an important risk factor in T1D.

In order to better understand why rs592645 is the best SNP for predicting copy number variation in *KIR3DL1/3DS1*, we used BLAT [22] to match the probe sequences of rs592645 on ImmunoChip against the allelic sequences of all KIR genes available from the Immuno Polymorphism Database [23]. Interestingly, we found that the SNP probes do not target *KIR3DL1/3DS1* but instead bind uniquely to the fifth intron of *KIR2DL4*, a neighbouring framework gene. Examining the *KIR2DL4* alleles matched by the rs592645 probes, we discovered that the SNP probes are in fact picking up copy number variation of KIR2DL4*005, an allele of *KIR2DL4* that undergoes copy number variation along with *KIR3DL1/3DS1*[24]. This explains the small but persistent misclassification error rate of 2% since our imputation is based on linkage disequilibrium between rs592645 and *KIR3DL1/3DS1* rather than on perfect discrimination between our target genes. We have identified 27 samples, which we believe, are consistently misclassified due to imperfect linkage disequilibrium (Additional file 1: Table S6).

We have observed other SNPs with more than three clusters that may correlate with copy number of other KIR genes and, given the availability of qPCR results, could be imputable in a similar manner.

## Abbreviations

CNV: Copy number variation; HLA: Human Leukocyte Antigen; KIR: Killer Immunoglobulin-like Receptor; knn: k-nearest neighbour; LOOCV: Leave-one-out cross-validation; qPCR: Quantitative polymerase chain reaction; SNP: Single nucleotide polymorphism; T1D: Type 1 diabetes.

## Competing interests

The authors declare that they have no competing interests.

## Authors’ contributions

Conceived and designed the experiments: JA Todd. Designed the qPCR assays: JJ, WJ, JA Traherne and JT. Performed the qPCR experiments for *KIR3DL1/3DS1*: DJS. Performed ImmunoChip genotyping: SO-G, W-MC, PC and SSR. Prepared data: HS, NMW, HG and OSB. Statistical analysis: NP, CW, JMMH and HS. Wrote the manuscript: NP and CW. Reviewed and revised the manuscript: JMMH, DS, JA Traherne, NMW and JA Todd. All authors have read and approved the manuscript.

## Supplementary Material

Additional file 1 Supplementary figures and tables. Click here for file
