# Supplementary material for: A hybrid qPCR/SNP array approach allows cost efficient assessment of KIR gene copy numbers in large samples
Source: BMC Genomics. 2014 Apr 11;15:274. doi: 10.1186/1471-2164-15-274 (PMC4029094; doi:10.1186/1471-2164-15-274)
Supplement: Additional file 1 — Supplementary figures and tables. [file 1471-2164-15-274-S1.PDF]

Supplementary

Figures

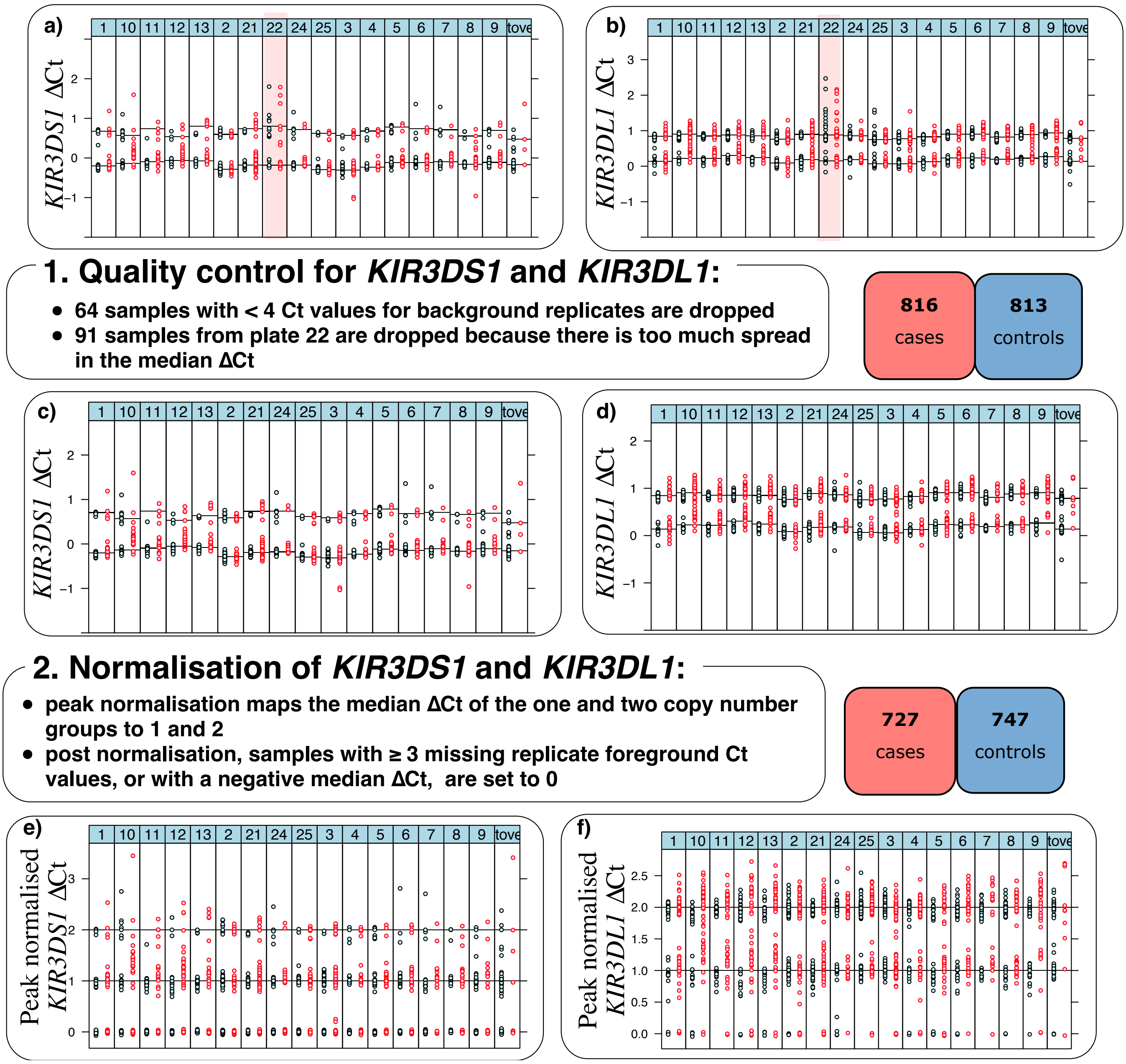

Supplementary Figure 1. qPCR plate effect on individual *KIR3DS1* and *KIR3DL1* ΔCt distributions. *KIR3DS1* and *KIR3DL1* ΔCt values for cases (red) and controls (blue) per qPCR plate. Negative ΔCt below −2 are not displayed for pre and post QC so as to better visualise the one and two copy number groups. Before QC, there are 1629 unique samples, 816 cases and 813 controls, arrayed over 18 plates with a maximum of 96 samples per plate. The QC involves two steps. In the first step, samples with less than four *STAT6* Ct values are dropped. In the second step, plate 22 (highlighted in red), which appears as the noisiest for both *KIR3DL1* (a) and *KIR3DS1* (b), is dropped. After QC, there are 1474 unique samples remaining, 747 cases and 727 controls, arrayed over 17 plates (c and d). Normalisation consists a linear transform which maps the medians of the one and two copy groups from each plate to 1 and 2 (e and f). After normalisation, negative ΔCts values are assigned to zero.

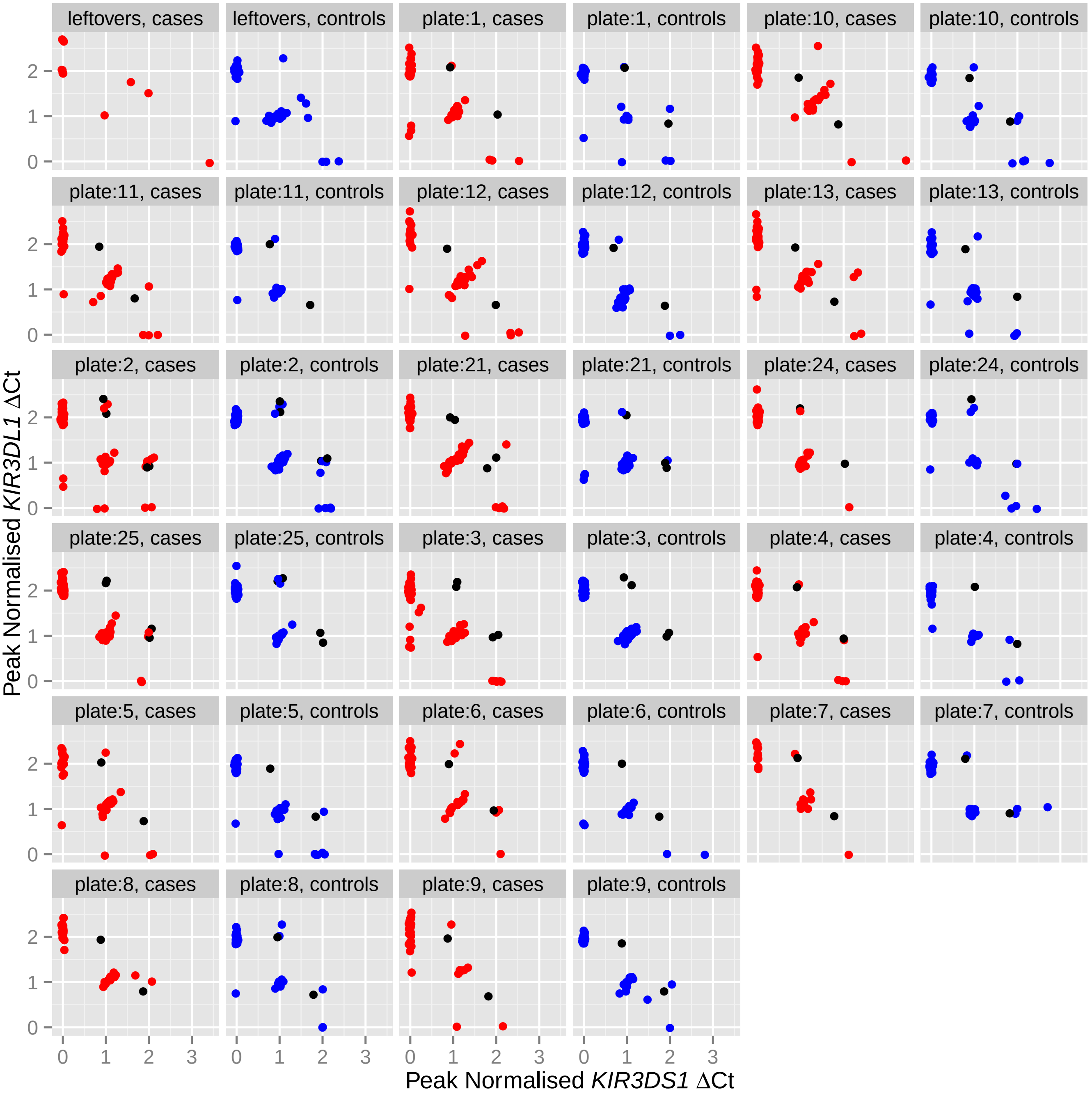

Supplementary Figure 2. qPCR plate effect on joint *KIR3DS1/3DL1* ΔCt distribution. Post-QC *KIR3DS1* and *KIR3DL1* ΔCt values for cases (red) and controls (blue) are plotted separately for each qPCR plate. The samples with known *KIR3DS1*-*KIR3DL1* copy number, 2-1 and 1-2, are plotted in black. We can see that there is a larger spread in cases than in controls which is especially marked in the 1-1 copy number group. Also, it is apparent that the ΔCt of *KIR3DS1* and *KIR3DL1* are correlated in the 1-1, 2-1 and 1-2 groups. We exploit this correlation in the copy number calling by doing bivariate clustering (see Methods).

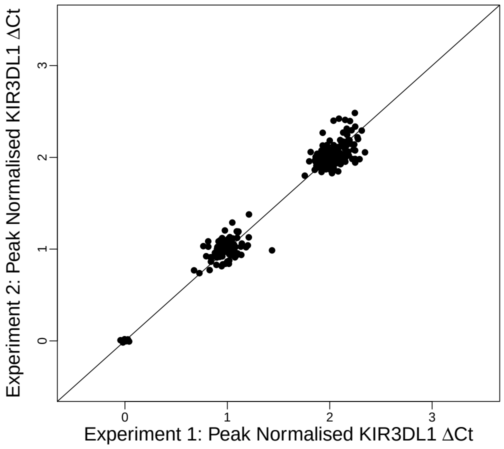

(a) Repeatability of *KIR3DL1* ΔCt post normalisation and QC ($r^2 = 0.961$).

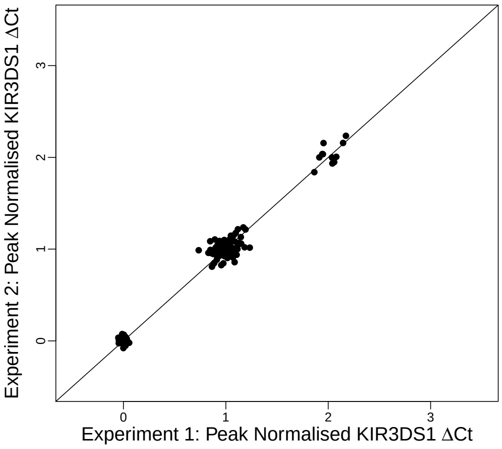

(b) Repeatability of *KIR3DS1* ΔCt post normalisation and QC ($r^2 = 0.99$).

Supplementary Figure 3. Repeatability of *KIR3DL1* and *KIR3DS1* ΔCt after QC and normalisation. In order to assess the reliability of the qPCR assay, 310 samples were re-analysed. We found very high reproducibility of the ΔCt values ($r^2 > 0.96$) confirming the reliability of our qPCR assay.

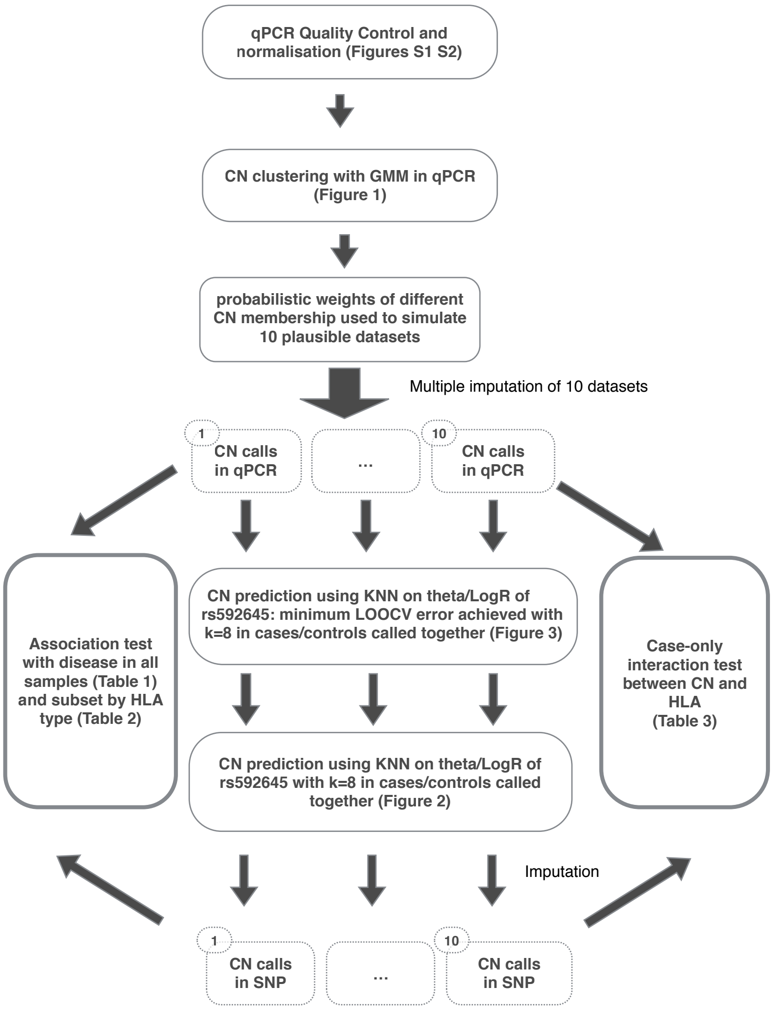

Supplementary Figure 4. Flow chart summarising the key steps involved in the k-nearest neighbour (KNN) imputation of KIR3DL1/3DS1 copy numbers (CN) in SNP data (LogR and Theta signal) from qPCR CN predictions obtained from Gaussian mixture models (GMM) clustering. GMM clustering of the qPCR data assigns to each sample a posterior probability of belonging to each CN group. Using these probabilities we can allow for the uncertainty of the CN calling when testing for association with disease by using multiple imputation. Multiple imputation involves the simulation of datasets (in this case, ten) from the probabilities returned by the GMM. We find that the SNP, rs592645, and k=8 minimises the LOOCV error rate. Association tests are conducted on each imputed dataset and inference combined using methods in `R` package mice (van Buuren and Groothuis-Oudshoorn, 2011).

Tables

Epitope		Residues (77-83)	HLA-B	HLA-A
HLA-Bw4	80I	NLR**I**ALR	B*1516 B*1517 B*1524 B*2702 B*3801 B*4901 B*5101 B*5108 B*5201 B*5301 B*5302 B*5701 B*5702 B*5801	A*2301 A*2402 A*2403 A*2407 A*2501 A*3201
HLA-Bw4	80T	DLR**T**LLR<br>SLR**T**LLR<br>NLR**T**ALR	B*1302 B*2701 B*2704 B*2705 B*3701 B*3802 B*4402 B*4403 B*4404 B*4405 B*4414 B*4417 B*4429 B*4435 B*4701	
HLA-Bw6		SLR**N**LRG	B*702 B*703 B*705 B*706 B*708 B*710 B*716 B*726 B*801 B*1401 B*1402 B*1501 B*1503 B*1504 B*1505 B*1507 B*1508 B*1509 B*1510 B*1514 B*1515 B*1518 B*1539 B*1801 B*3501 B*3502 B*3503 B*3508 B*3901 B*3906 B*3928 B*4001 B*4002 B*4006 B*4011 B*4023 B*4101 B*4102 B*4202 B*4501 B*4601 B*4801 B*5001 B*5002 B*5501 B*5601	

Supplementary Table 1. *HLA-A* and *HLA-B* alleles which carry the serological epitope HLA-Bw4 can be further subdivided as HLA-Bw4-80I or HLA-Bw4-80T, depending on whether the amino acid at position 80 in the heavy alpha chain of the HLA Class I protein is an isoleucine (I) or a threonine (T) (Gumperz et al, 1997; Martin et al, 2002).

Gene	Oligos	Sequence (5'-3')
KIR3DS1	Forward Primer	`CATCGGTTCCATGATGCG`
	Reverse Primer	`GGGAGCTGACAACTGATAGG`
	Probe	`AACAGAACCGTAGCATCTGTAGGTCCCT`
KIR3DL1	Forward Primer	`CACAGTTGGATCACTGCGT`
	Reverse Primer	`CCGTGTACAAGATGGTATCTGTA`
	Probe	`CCCTTCTCAGAGGCCCAAGACAC`
STAT6	Forward Primer	`CCAGATGCCTACCATGGTG`
	Reverse Primer	`CCATCTGCACAGACCACTCC`
	Probe	`CTGATTCCTCCATGAGCATGCAGCTT`

Supplementary Table 2. The qPCR primers. These were originally designed by Jiang et al (2012).

HLA Epitope	Cases	Controls	Total
N/A	3822 (11)	2681 (70)	6503 (81)
HLA-Bw6	1175 (308)	753 (199)	1928 (507)
HLA-Bw4-80T	651 (162)	754 (174)	1405 (336)
HLA-Bw4-80I	1096 (266)	1174 (284)	2270 (550)
HLA total	2922 (736)	2681 (657)	5603 (1393)

Supplementary Table 3. HLA epitope classification of subjects in study. In parentheses, number of subjects analysed with qPCR post QC. No HLA typing was available for the N/A category. The HLA epitopes are defined in Table 1. An individual is assigned to an HLA epitope group if he is a carrier of at least one allele of that group. So that each individual only belongs to a single HLA epitope group, the assignment priority is first HLA-Bw4-80I, then HLA-Bw4-80T and finally HLA-Bw6 allele if no HLA-Bw4 alleles were found.

Name	Position	SNP	QC	p-value θ	p-value R
seq-rs597598	60007252	[A/G]	ok	3.19E-03	7.81E-01
seq-rs598452	60007428	[A/G]	ok	6.53E-01	2.62E-01
seq-t1d-19-60007809-C-G	60007809	[G/C]	ok	3.64E-02	6.27E-06
seq-rs55761930	60008141	[T/C]	ok	6.33E-01	6.12E-01
seq-rs10500318	60012591	[A/G]	ok	7.59E-11	1.31E-13
seq-rs592645	60012739	[A/T]	ok	8.85E-01	3.38E-09
seq-rs604077	60013208	[A/G]	ok	4.82E-03	1.20E-01
seq-rs604999	60013409	[A/G]	ok	1.77E-15	9.99E-04
seq-t1d-19-60014013-A-C	60014013	[T/G]	lowcallrate	8.74E-01	3.15E-08
rs3865507	60014188	[T/G]	ok	8.62E-03	6.93E-17
seq-rs3865510	60016051	[A/C]	ok	2.23E-10	2.04E-10
seq-rs648689	60016286	[A/G]	ok	2.31E-01	1.03E-02
seq-rs649216	60016447	[T/C]	ok	2.85E-02	1.04E-13
rs581623	60018551	[A/G]	ok	3.76E-02	2.06E-13
seq-rs4806568	60022568	[A/G]	lowcallrate	1.44E-20	2.93E-01
seq-rs674268	60024002	[T/C]	lowcallrate	1.43E-02	2.90E-01
rs12461010	60024413	[A/G]	ok	4.72E-01	1.72E-01
seq-rs2295805	60028513	[T/C]	lowcallrate	9.55E-08	8.40E-04
seq-rs12976350	60030391	[T/C]	lowcallrate	1.70E-05	5.07E-01
seq-t1d-19-60034052-C-T	60034052	[A/G]	hwe	3.27E-02	4.07E-01
rs4806585	60038236	[T/G]	hwe	2.20E-11	2.42E-02
seq-rs62122181	60039178	[T/C]	lowcallrate	2.40E-13	2.26E-01
rs10422740	60052298	[T/C]	monomorph	7.78E-01	8.49E-02
rs640345	60054671	[A/G]	ok	3.61E-07	6.83E-02
seq-t1d-19-60054973-T-C	60054973	[A/G]	ok	2.92E-01	2.28E-04
seq-t1d-19-60056605-A-T	60056605	[A/T]	ok	3.99E-01	1.48E-16
seq-t1d-19-60056721-C-T	60056721	[A/G]	ok	9.02E-01	2.04E-09
seq-rs10407958	60063974	[T/A]	ok	1.06E-02	5.45E-10
seq-rs1654644	60065174	[T/G]	ok	7.94E-14	5.21E-12
rs3826878	60069023	[A/G]	ok	2.63E-05	3.55E-06

Supplementary Table 4. The 30 ImmunoChip SNPs which fall in the *KIR3DL1* region according to build36/hg18, nineteen of which are significantly associated with *KIR3DL1/3DS1* copy number (highlighted in blue).

KIR3DS1-KIR3DL1 Copy Number	Jiang et al (2012)	dataset SNP	qPCR
0-2	57.95	60.43	60.38
1-1	30.65	30.38	29.58
2-0	4.07	3.75	3.66
1-2	2.26	1.42	1.83
0-1	2.18	1.59	1.63
2-1	1.81	1.83	2.10
1-0	0.58	0.38	0.47
3-0	0.32	0.21	0.34
0-3	0.10		
3-1	0.02		
2-2	0.02		
0-0	0.02		
4-0	0.01		
1-3	0.00		
0-4	0.00		

Supplementary Table 5. Comparison of *KIR3DS1-KIR3DL1* genotype frequencies calculated from Jiang et al (2012) KIR haplotype frequencies (assuming Hardy-Weinberg) compared to those obtained from our qPCR and SNP dataset.

	KIR3DS1-KIR3DL1 qPCR Copy Number	qPCR posterior	KIR3DS1-KIR3DL1 knn prediction
1	0-1	1.00	0-2
2	0-1	1.00	0-2
3	0-2	1.00	0-1
4	0-2	1.00	1-1
5	0-2	1.00	1-1
6	0-2	1.00	1-1
7	0-2	1.00	1-1
8	0-2	1.00	1-1
9	1-0	1.00	1-1
10	1-0	1.00	1-1
11	1-0	1.00	2-0
12	1-0	1.00	2-0
13	1-1	1.00	0-2
14	1-1	1.00	0-2
15	1-1	1.00	0-2
16	1-1	1.00	2-1
17	1-2	1.00	1-1
18	1-2	1.00	1-1
19	2-0	0.97	2-1
20	2-1	1.00	0-2
21	2-1	1.00	1-1
22	2-1	1.00	1-1
23	2-1	1.00	1-1
24	3-0	1.00	2-0
25	3-0	1.00	2-0
26	3-0	1.00	2-0
27	3-0	0.98	2-0

Supplementary Table 6. These 27 samples are consistently misclassified by leave-one-out knn imputation whereas they are called with confidence in the qPCR since the posterior is close to 1. We believe these errors are due to the imperfect linkage disequilibrium between rs592645 and *KIR3DL1/3DS1*.

References

Gumperz JE, Barber LD, Valiante NM, Percival L, Phillips JH, Lanier LL, and Parham P. 1997. Conserved and variable residues within the Bw4 motif of HLA-B make separable contributions to recognition by the NKB1 killer cell-inhibitory receptor. *J. Immunol.* **158**: 5237–5241.

Jiang W, Johnson C, Jayaraman J, Simecek N, Noble J, Moffatt MF, Cookson WO, Trowsdale J, and Traherne JA. 2012. Copy number variation leads to considerable diversity for B but not A haplotypes of the human KIR genes encoding NK cell receptors. *Genome Res.* **22**: 1845–1854.

Martin MP, Gao X, Lee JH, Nelson GW, Detels R, Goedert JJ, Buchbinder S, Hoots K, Vlahov D, Trowsdale J, et al. 2002. Epistatic interaction between KIR3DS1 and HLA-B delays the progression to AIDS. *Nat. Genet.* **31**: 429–434.

van Buuren S and Groothuis-Oudshoorn K. 2011. mice: Multivariate imputation by chained equations in r. *Journal of Statistical Software* **45**: 1–67.
